# For a more-than-human public health

**DOI:** 10.1057/s41292-020-00210-8

**Published:** 2020-10-21

**Authors:** Janina Kehr

**Affiliations:** Institute of Social Anthropology, University of Bern, Lerchenweg 36, 3012 Bern, Switzerland

**Books Reviewed**

Cassidy, A. (2019) *Vermin, Victims and Disease: British Debates over Bovine Tuberculosis and Badgers*. London: Palgrave.

Keck, F. (2020) *Avian Reservoirs: Virus Hunters and Birdwatchers in Chinese Sentinel Posts*. Durham, NC: Duke University press.

The “more-than-human” has become an indispensable perspective in science and technology studies and in the social study of the life sciences more widely. In general, the more-than-human indicates that being, doing, living and relating is shaped not only by human but also by non-human worlds, materials and entities—be they animals, technologies, microbes or elements. This perspective takes multiple forms. Some scholars are influenced by studies on Actor Network Theory and, more recently, on “modes of existence” (Latour 2012) in the Anthropocene. Others are influenced by work on ontologies of practice (Mol 2002), in which science and medicine are conceived as coming to existence by means of coordination and relation between humans, technologies and knowledges (Berg and Mol 1998; Mol 2002). Others emphasize new materialist approaches (Haraway 2016), particularly in feminist science studies, which aim to register “life in capitalist ruins” (Tsing 2015) where cross-species relations with critters and plants come to centre stage. Still others locate themselves within scholarship on “matters of care” (Puig de la Bellacasa 2017) and “ecologies of support” (Duclos and Criado 2020) that pushes feminist questions on the reproduction of life into ecological and more-than-human terrains. Finally, some look to ethnographic studies in indigenous spaces in the Amazon and Australia, which aim to provincialize modern Euro-American “life/non-life” distinctions (Povinelli 2016) and nature–culture dichotomies (Descola 2013), not least through the study of human–animal relations.

The last topic, the human and the non-human animal, is at the heart of two recent books on zoonoses—that is, infectious diseases that can be transmitted from animals to humans. One study is anthropological (Frédérick Keck’s *Avian Reservoirs*), and the other is historical (Angela Cassidy’s *Vermin, Victims and Disease*). In this time of the COVID-19 pandemic, both books are tragically timely investigations into the scene of more-than-human public health, where animals, microbiologists, public health officials, veterinarians, politicians, pathogens, non-governmental organizations, sovereign territories and natural environments are deeply entangled, and in which scientific uncertainties and power-relations loom large.

In *Avian Reservoirs,* Keck proposes a theoretical distinction between preparedness and prevention^1^ in the management and anticipation of avian influenza in Hong Kong, Taiwan and Singapore, to understand “how technologies to prepare for influenza pandemics have transformed our relations to birds” (p. 2). Through a methodological mix of what he calls “fieldwork in philosophy” (p. 6), as well as ethnography among virologists, birdwatchers, veterinarians and public health officials, Keck shows how far the anticipation of pandemics “has modified the world in which humans live with animals” (p. 2). This is a world in which the industrial increase of livestock for human consumption comes with new pandemic risks, as well as locally and politically specific techniques that mitigate those risks and weaken their potentially lethal outcome for humans and animals alike. What makes Keck’s comparison of pandemic preparedness in different Asian settings remarkable is that the three sites are not only more-than-human “reservoirs” for avian zoonoses, but are also disputed political territories bordering the People’s Republic of China. All are haunted by unclarified relations with the world’s most populous country, thus foregrounding questions of sovereignty, transparency and ecological interdependency, which are of course also present in the investigation and management of emerging infectious diseases. Keck thus shows how far all three territories fashion themselves as public health outposts—Hong Kong as a “sentinel post” (ch. 4), Singapore as a centre for “simulations” (ch. 5) and Taiwan as a site of “stockpiling” (ch. 6), each thereby finding its own route to sovereign pandemic preparedness next to its powerful neighbour.

In contrast to Keck’s vast and sometimes dizzying diversity of fields and theoretical arguments, Angela Cassidy carves out a fine-grained, more-than-human history of a particularly British scientific controversy at the crossroads of human health, animal health and environmental conservation. Cassidy’s focus is “the badger/bTB knowledge controversy” (p. 30), i.e. the question of whether or not badgers are a reservoir for bovine tuberculosis (bTB), which is a zoonotic disease that mainly affects cattle but can also affect humans in the form of tuberculosis disease. Following scientific experiments, media debates and controversial policies around the slowly developing mycobacterium *M. bovis* from the 1960s to the present, Cassidy traces the role of diverse forms and contexts of human–animal relations in the making of science and policy in the UK, where ideas about agriculture, landscape and rurality intersect with questions of animal care, large-scale livestock production and intensive farming. Cassidy argues that the “Great British Badger Debate” (p. 23) that has developed around bovine TB is indeed something very British, as it revolves around some of the “Big Questions” that move citizens in that country, namely “how do and how should people live alongside other animals? What does it mean to care (…)? What is the proper relationship between science and policy?” (p. viii). Throughout the book, Cassidy shows how knowledge is gradually built and revised through oftentimes highly politicized controversies between different epistemic communities: farmers and veterinarians, pest control scientists and field biologists, and conservationists and animal advocates. All practice different “cultures of care” (p. 17), in which the agency of human and non-human organisms is under moral, political and scientific dispute.

The two books take microbes as a starting point: bird flu viruses for Keck and tuberculosis mycobacteria for Cassidy. The questions and theoretical framings with which the two authors approach the two pathogens differ as much as the microbes in question, however. Cassidy follows a rather classical history of science approach to study a national knowledge controversy, by foregrounding the everyday practices and scientific points of view of different epistemic communities and the uncertainties surrounding knowledge production. One of her aims is to imagine how conflict-laden science could inform policy in knowledge societies in more participatory and dialogical ways, if uncertainty and revocability were publicly agreed to be inherent features of scientific practice. Keck’s work, by contrast, is nourished by French structuralist anthropology as well as by the history of ideas. With heavy theoretical baggage at hand, where semiotic theory is crisscrossed with ontologies of hunter-gather societies and Foucauldian biopolitics, Keck aims to understand pandemic preparedness “at the animal level” (p. 3) by attending to the “relations between humans and their environment” (p. 6)—in other words, by inquiring into how such relations shape scientific results and public health approaches. Despite the two authors’ highly different angles and aims, they come to a surprisingly similar conclusion: the necessity of envisioning a scientifically informed, more-than-human public health. Keck does so through an anthropological bricolage, and Cassidy through domestic historiography.

## Pandemic preparedness, anthropological *bricolage* and a science of signs

Keck conceives of “avian reservoirs” (the environments in which birds or other animals potentially carry novel viruses like SARS-CoV-2 and transmit them to humans) as an Amazonian forest, “a space where human and nonhuman animals are connected” (p. 4). He thereby steps in the intellectual footsteps of French anthropologist Claude Lévi-Strauss, whose notion of the *penseé sauvage* (“the savage mind”), inspired by ethnographic research in the Amazon, was the topic of Keck’s first book^2^. In *Avian Reservoirs*, Keck conceives of birds as signs or sentinels, which have the ability to signal disease and ecological change to humans, especially in times of environmental destruction and mass-livestock production. Building on Lévi-Strauss’s “cynegetic” notion of the social (cynegetic essentially means something related to hunting), Keck explores Asian preparedness as a meaningful public health technology, based on hunting relationships between humans, birds and viruses. Keck defines this cynegetic social with Lévi-Strauss as “a series of signs produced in a situation of communication between hunter and prey” (p. 27).

In Keck’s work, the cynegetic social indicates that prey (birds and their microbes) and hunters (microbiologists and ornithologists) are deeply interconnected in “sentinel posts” (natural territories or labs) in Asia, where they communicate with one another. For Keck, this form of communication in a hunter-gatherer mode stands as an alternative to knowledge more commonly present in Euro-America, which he sees as based on a nature–culture divide that is unprepared to imagine—as he put it in an interview—“what’s happening to us”.^3^ In his Asian field sites, scientists, or hunters, come to know their prey through signs or signals that prey, in the form of birds or viruses, sends while flying, migrating, mutating or simply existing. A sign can be a viral mutation in a genetic sequence perceived through the technological analysis of vast amounts of genetic information stored in databanks, or it can be a changed migratory flyway inscribed on a map in a natural reserve, to give just two examples. By getting to know prey in such a “cynegetic” way, according to Keck, microbiologists and ornithologists collect samples and signals to “monitor pathogens to anticipate a pandemic” following an “animistic ontology” (p. 28). This is what Keck subsumes under preparedness. Preparedness, in this definition, is ultimately a science of signs, which tries to foresee the future by understanding the diverse signals that the past and present of microbial or animal worlds transmit to humans. Microbes, human and non-human animals are thus intimately connected in their fate. By contrast, when microbiologists or veterinary scientists produce statistics “on the paths of contagion to justify the intervention of the state”, as in flu outbreaks monitored in what Keck calls “sentinel chickens”^4^ (p. 70) on farms, they follow an “analogistic ontology” and thus a “pastoralist technique” (p. 28)—that is, an approach “to regulate the proliferation of beings through a sovereign gestures of sacrifice” (p. 27). Keck writes: “If prevention excludes the perspectives of animals on public health management under a sacrificial rationality underlying culling and vaccinating, preparedness includes them by extending participation through techniques of monitoring” (p. 12). The preventive culling of millions of flu-infected chickens on animal farms, in order to preserve humans’ health, is one such sovereign gesture that sacrifices animals in the name of human health. At play, for Keck, is a biopolitical form of pastoral care in the Foucauldian sense, governing human health through state intervention in animal and human lives, separating animal and human worlds rather than seeing them as shared and interconnected.

The diverse historical and ethnographic examples that figure in Keck’s book (ranging from outbreak simulations in hospitals to ornithological lures in nature reserves; from vaccine development in the lab to mass-livestock production on chicken farms; from Durkheimian sociological thought to conservation practices in museums) are the empirical scenes on which Keck philosophizes about preparedness and prevention as two different modalities of public health, or rather as two different worlds of pandemic response. While Keck conceptualizes preparedness as a hunting technique working through the perception of signs in nature that may have gotten lost in “industrial food production” (p. 174), he sees prevention as a pastoral technique that relies on modern state intervention and “industrialized-administrative ways of thinking” (p. 174). Preparedness figures as an almost idealized, more-than-human public health that valorizes animals as beings deeply entangled with humans’ fate, while prevention comes along as a powerful facet of modern biopolitics, where animal well-being is subordinated to human development and economic growth.

This implicit valuation of Keck’s version of preparedness has an ethnographic root. During his research in microbiological research institutions and museums, as well as among ornithological societies in Hong Kong, Singapore, Taiwan and Paris, Keck has come to “share birdwatchers’ passion for bird species and microbiologists’ curiosity for viral mutations” (p. 3), a fascination that is felt throughout the book. It is maybe this passion for the science of birds, viruses and classification, combined with his initial training as a philosopher of structural anthropology, that has lead Keck to become fascinated by preparedness as a science of signs and human–animal relations *in* public health, in an ontological fashion, rather than with the everyday facets *of* public health, in the face of an animal disease, as one might expect of an ethnographically working anthropologist. While some classical public health techniques like vaccine development and the stockpiling and distribution of antiviral treatments (chap. 6) or the simulation of emergency outbreaks in hospitals (chap. 5) are under scrutiny in the book, Keck does not intend to understand them in their contradictory everydayness as such. Rather he incorporates them into his own structural and/or philosophical interest about prevention and preparedness as two ontologically different worlds of human–animal relations. In this sense, Keck is a true Levi-Straussian *bricoleur*, a figure he luminously analysed in his first book. A bricoleur “uses any operators at hand, whose uses he redefines according to the occasions at hand” (Keck 2004, p. 34). As a *bricoleur*, I argue, using Keck’s own words from 2004, Keck is part of “an entirely meaningful world, whose signs he arranges otherwise so as to produce novel significations^5^” (pp. 34–35). In *Avian Reservoirs*, novel concepts and displaced meanings indeed abound. In Keck’s highly creative arrangements, an understanding of what more-than-human prevention and preparedness actually *are* in specific places is nevertheless covered by a thick forest of theory, which excels in conceptual work but unfortunately obscures a narrative on the tangible stakes of more-than-human global public health. Keck himself defines his conceptual work as “critical”, aiming to “make a difference in debates that are often confused about pandemic preparedness and thus open alternatives to securitizing views of relations between humans and their environment” (p. 7). I am highly sympathetic to this definition of critique as an exercise in conceptual displacement as well as to Keck’s insistence on understanding pandemic preparedness not only as a practice of public health in an all-too-human biosecurity framework, but as more-than-human public health. As an anthropologist of public health, however, I am also frustrated, as Keck’s approach seems to be more defined by a desire to think *with* rather than *about* the relation between public health techniques and human–animal relations. This comes at a cost.

For example, there is surprisingly little explicit debate in the book about the unspoken hierarchy between human and non-human health in capitalist economies, a topic that nevertheless lingers on many of the book’s pages. An analysis of the political economy of animal production in Asia and beyond—be it for food or for the lab—is thus barely present, despite some sparks of insight, specifically in chapter four, when Keck talks about animal farms as “metabolic factories” (p. 75) and focuses on the “bad karma” (p. 81) of mass animal consumption as seen through the eyes of a Buddhist association. Some figures and facts on global livestock production could have been enriching. In addition, in-depth descriptions of avian flu science and pandemic control in Asia are certainly at centre stage in the book, but the constant jumping between anthropological conceptualization and viral facts, the bricolage between ethnographic observation and historical description from intuitively unrelated fields, as well as the multitude of theoretical arguments and geographical locations make it difficult to follow. This “shapeshifting approach” (Lau 2020, p. 2) almost prevents the reader from distilling the book’s key messages about global public health. For example, it obscures the importance of envisioning global health as One Health, where the conditions of life of animals and their caretakers are seen as vitally important in the management of newly emerging infectious diseases (p. 177). That slaughterhouses worldwide are hotspots of the COVID-19 pandemic thus comes as almost no surprise, as they are sites of intense exploitation of (migrant) labour and of animal bodies, where capitalist profit is extracted from an intentional neglect of human and animal health. Finally, to readers not initiated in Keck’s theoretical and philosophical background, intelligibility can be a real issue throughout the chapters. Was it not for the clearly written passages of the conclusion, I admit that I would have been lost more than once in Keck’s conceptual Amazonian forest.

## Domestic historiography, public knowledge controversies and uncertain science

Angela Cassidy’s *Vermin, Victims and Disease*, by contrast, is written in a more straightforward, perhaps even didactic manner, and follows a completely different path into the science of animal disease. As a historian of science and science–policy relations, Cassidy takes a chronological and localized approach to zoonoses. While she construes her investigation around animals as disease “reservoirs” (p. 3), as Keck does, Cassidy’s aim is to provide a thick historiographical account of the scientific and political debates that have developed around bovine tuberculosis (bTB) in Britain, with badgers identified as the mycobacterial reservoir. Cassidy’s country-based historical approach allows her to thoroughly detail the political and scientific stakes of the controversy’s multiple turning points, and link it to national history, governmental politics, cultural sensibilities and the “framing” (p. 276) of disease in the UK.

Unlike newly emerging viral diseases, bTB has been a continuous issue in British public health since the beginning of the twentieth century. It is as much a “chronic agricultural problem” for farmers as it is “an environmental risk (…) to fragile wildlife and ecosystems” (p. 4). Questions of chronicity and latency are vitally important, but so are political and scientific framings and economic stakes. Currently, the British Government spends around 100 million pounds each year on the effects of bovine Tb and has done so throughout the last decades; bovine TB has been the object of costly investigations, trials and preventive measures in laboratories and field sites in Britain since the 1960s. As such, it has become the object of an intense “public knowledge controversy” (p. 14), highly shaped by media coverage and political as well as economic debates in the country.

Through a careful analysis of her historical material, Cassidy describes how bTB, which developed from a “well-controlled disease” to a “resurgent, poorly understood epidemic” (p. 12), turned into one of the central animal policy issues in Britain, where animal health, human health and conservation interests intersect (p. 12). When bTB came to be gradually differentiated from human tuberculosis medically in the first part of the twentieth century—it mainly affected cattle—it was framed as a zoonosis. As such, it was relegated to the domain of veterinary science by the mid-twentieth century, a disease to be “stamped out” through government action. It is only in the 1970s that bTB became a troubled public affair again, when the badger, a historically both loved and hated animal in Britain, entered the scene as a potential vector of disease for animals and humans alike. At the time, theories of disease ecology were becoming popularized, a more politicized approach to wildlife conservation had developed, and social justice and environmental movements were growing in Britain and elsewhere. Ecological approaches saw “microbes as active elements of dynamically changing ecological systems” (p. 277). Bovine TB, meanwhile, came to be increasingly seen as an “environmental disease” (p. 277) beyond farmers’ and veterinarians’ scope, and thus something to be managed by the country’s pest control officers. At the same time, badger protection campaigners lobbied in Parliament against the culling of badgers that pest control advised, creating a strong opposition to government policy, which persists today also in the name of wildlife protection and ecological conservation. It is here that an interesting parallel to Keck’s stories of disease ecology and microbe reservoirs in Asia can be found. For birdwatchers in Hong Kong’s Pearl River Delta, where migratory flyways of wild birds are situated, birds are not only sentinels for flu outbreaks, but also, like Cassidy’s badgers, “sentinels for the environment” (Keck, p. 87), indexing changes in biodiversity as well as “environmental threats affecting birds and humans alike” (Keck, p. 89).

In the mid-1990s, a further turning point took place, which allows Cassidy to shift attention from the gradual making and unmaking of scientific facts across different epistemic communities, to the role of media in highly unsettled knowledge controversies. The badger-bTB controversy became an increasingly “frontstage” (p. 241) policy problem, fiercely debated not only in government-commissioned expert reports but also in the mass media, testifying to the emergence of “evidence-based policy” (p. 220) in political life in Britain at the time. While bTB policy in the 1970s had been shaped by enrolling a wider public in the policy process, ranging from naturalists, farmers, ministries, and wildlife experts, this strategy was gradually abandoned, mainly due to cost, thereby eroding “structures for direct engagement” (p. 266). The void of participation was increasingly filled by mass media coverage, which removed “more subtle forms of negotiation and communication” (p. 266). The consequence was an ever more polarized and partisan debate, that circled around the culling (or not culling) of badgers.

The “sudden explosion” of badger/bTB into mainstream media since the late 1990s testifies to the “transformation in mass-media industry” (p. 262) in the UK, including its increasing commercialization, the importance of news value and the frequency of media storms. With this transformation, a shift in science–policy relations occurred, as both science and policy were now highly shaped by media coverage and its breath-taking speed. Of course, what Cassidy describes here could not hold more true for the current COVID-19 pandemic and its incessant, highly mediatized scientific debates.

Throughout her book, Cassidy shows how much the history of bTB mirrors a “broader reconfiguration of the domains of human and animal health since the late nineteenth century” (p. 276), a domain shaped by diverse scientific practices and changing science–policy–media relations. By taking a “symmetrical stance” (p. 15) on the Btb/badger public knowledge controversy, inspired by David Bloor’s “strong programme” (and thus proceeding without judging about truth or falseness), Cassidy truly illuminates “all sides of the debate” (p. 15) and its manifold actors. This perspective also includes more-than-human actors in an Actor Network Theory fashion, taking animals, microbes and chemical substances seriously. While firmly assuming a national or domestic history approach, staying within Britain’s borders, Cassidy nevertheless goes beyond the boundaries of a human historiography by demonstrating how the bTB/badger debate has been shaped by more-than-human worlds (p. 47). Unlike Keck, Cassidy does not pretend to take on animals’ perspectives, however. Rather, she examines animals’ “traces” (p. 18), particularly those of badgers and cattle, left in the historical documents she analyses, be those traces in media coverage, photographs, drawings in children’s’ books, reports of field officers of the Ministry of Agriculture, Fisheries and Food, veterinarian expertise, farmers’ complaints, or policy and campaigning documents.

By tracing the history of a scientific controversy in this way, Cassidy aims to create “better public and institutional memories of a notoriously ‘intractable’ policy problem” (p. 13), a more-than-human problem that might never be solved. She is animated by a deep concern for “how science, technology and medicine interact with policy and the public sphere” (p. 13). Through her case study, Cassidy shows how science is made up of different epistemic communities and cultures of care. This allows her not only to lay out the multiplicity that characterizes the making of disputed scientific knowledge, but also the uncertainty and refutability of Science, especially in complex ecological terrain. She thereby effectively deconstructs the idea of “Science” writ large as an “authoritative resource” that can “simply provide ‘all the answers’” (p. 275). This simultaneously very modern and rather old idea of science, coming straight out of the enlightenment, seems to have more traction than ever, despite incessant attempts from within science studies to deconstruct it. Such a deconstructive take on science, even if not the most innovative, continues to be highly relevant for current controversies in public health, as the current COVID-19 debate demonstrates^6^. The dazzling speed of change and refutability of scientific facts, coupled with a firm belief in them as taken-for-granted evidence or eternal truth, and then combined with rather little interrogation of knowledge and data production as such, is one of the startling paradoxes of our times. Cassidy grapples with this as a question of high “expectations” towards “The Science”, which are continuously raised and broken in incessant loops.

On this last point, Cassidy’s books strength is also its limitation. While being an extremely well-crafted historical account with clear relevance to public health controversies in the present, perspectives that go beyond this case study are largely left out. Cassidy might have dared to draw much more general conclusions about the making and unmaking of scientific expectations in the middle and long term, as well as to reflect on the modernist hauntings of Big Science with its promises to fix the problems of public health more generally. The tensions she raises are certainly not only exemplary of the bTB/badger debate, but index much larger questions of biological and public health latency in the face of political and economic urgency and the economic pressures of evidence-based policy. Even if tuberculosis, with its biology of slowness and latency, might indeed be a disease that carves out specific temporal tensions in public health (Kehr 2021), the omnipresence of media storms around infectious diseases, with epidemiological instant reporting and “real-time surveillance” (Engelmann 2020), makes it only more important to learn from “slow diseases” more broadly, and to speak out against the attraction of quick-fixes. So while Keck’s analysis could have profited from some less theoretical ambition to clarify the political stakes of zoonoses in global public health in a more intelligible manner, Cassidy’s descriptions could have gained in boldness via some more theoretical determination to conceptualize the science–policy nexus beyond the British bTB/badger debate, and thereby speculate on questions of scientific uncertainty and temporality in global public health. However, both books truly enrich the bourgeoning field of history and anthropology of zoonosis (Brown and Kelly 2014; Keck and Lynteris 2018; Kelly and Marí-Saéz Kelly and Almudena 2018; Lynteris 2019; Porter 2013; Woods et al. 2018), and allow us to see the importance of a more-than-human public health.

Indeed, each book in its own way is full of historical and anthropological details of the conflict-laden, more-than-human world of One Health, where the control of zoonotic diseases intersects with food safety in times of industrial livestock production; where concerns for nature conservation and species extinction are entangled with microbial science and public health surveillance; and where human health cannot be conceived outside of animal health and welfare. Taken together, the books reveal the complicated political trade-offs, intricate ecological tensions, and powerful medical measures of more-than-human diseases and measures of control that affect animals and humans alike. As zoonoses have been and will continue to be a vital feature of human and animal life on this planet, both books are timely resources to better understand the debate surrounding public health measures that grapple with the control of zoonoses, not least in the face of the ongoing COVID-19 pandemic.

## More-than-human public health

Let me close with a vignette: on June 6, 2020, the Dutch government ordered the culling of thousands of minks in animal farms in the south of the Netherlands. SARS-CoV-2 had been detected in farms that raise the animals for fur. The virus was most probably introduced by a farm worker who had COVID-19, that is, through human–animal transmission. The Dutch government feared that infected mink would become a novel “viral reservoir that could cause new outbreaks in humans”^7^ in return, through a reverse animal–human transmission. To date, such reverse transmission has not been proven, and is shrouded in scientific uncertainty. Thousands of mink were nevertheless killed.

Having read Cassidy and Keck, neither the novel virulence of species jumping pathogens originating from “animal reservoirs”, such SARS-Cov-2, nor the subsequent political order of culling, despite enormous scientific uncertainty, came as a big surprise to me. This “sacrificial public health strategy”, to speak in Keck’s terms, and its modern desire to control newly emerging pathogens, through which thousands of animals are killed in the name of disease prevention, is a consequence of a twentieth-century history of ecological change and mass-livestock production for human consumption and profit, which is now faced with its own biological looping effects. Rather than being an example of a science-led policy decision, culling can be understood, in light of Cassidy’s work, as a symbolic, almost desperate decision to perform political control in an uncontrollable situation. The many more-than-human effects of this “particular biology of modern history—the biology of twentieth century biopolitics” (Landecker 2016, 25), as historian biologist Hannah Landecker has called it, are met with an all-too-human political intervention. As Landecker points out, “the complex materiality of life adapting to management and manipulation at enormous scale well beyond the frame of human intention” (ibid., pp. 25–26) is in “us”, be it through food, disease or their pathways. From Keck and Cassidy we learn that it is in animals too.

It is for this reason that both books, despite all their differences, end with a similar call to envision what I have called a more-than-human public health throughout this essay, a vision of public health that includes microbes, humans, animals and the environment alike. Cassidy wants “historically awkward animals” like badgers to “flourish”, and calls for the “need to find modes of co-existence which can take account of animal agency and benefit all publics” (p. 288), beyond the fear of animals as disease reservoirs or “vermin”. She therefore suggests “experiments with dialogue and participatory governance” in politics (even if she does not include animals explicitly), where the “goal of consensus” (p. 289) needs to be set aside in favour of an acknowledgment of conflicting cultures of animal care, to find ways of working together. Keck contends that “improving biosecurity infrastructures means being attentive to the conditions of life of birds and those who take care of them, and sharing with equity the valuable products that come out of this interaction”. Not unlike Cassidy, he also calls for “the participation of all actors involved in the management of emerging pathogens, which compels experts to reframe concepts of causality relating humans, animals, and microbes” (p. 177). While Keck focuses on the interdependency of human and non-human animals in the management of pathogens, Cassidy focuses on the interrelation of science and policy in situations of environmental conflict. Both go beyond the “short temporality of emergencies”, as Keck calls it, to consider “the long temporality of ecologies” (p. 177). Cassidy does so by focusing on a slow disease, and Keck by taking on the point of view of birdwatchers.

Cassidy’s and Keck’s shared call for more inclusive and respectful human–animal relations in a world on the brink of ecological disaster and emerging diseases seems, unfortunately, more akin to a utopian desire than a realistic option. Nevertheless, their conclusions incite readers to develop a more-than-human “art of noticing” (Tsing 2015) of what is going on in the domain of public health, where longer political temporalities continue to intersect with complex disease ecologies and power-laden, economized forms of mass production of life and death. As such, both books are not only “diagnostic” of “social diseases on a planetary scale” (Wald 2020), but also call for a broadening of questions regarding disease manifestation, causation and treatment. In such a view, the specificity of diverse pathogens is as important as the “variability of their interactions with other species” (Brives 2020) in partly shared, yet hotly disputed, environments. Conceiving of global public health as more-than-human public health, I want to argue, has the potential to push the One Health approach further. One Health should go beyond the mere inclusion of animals and the environment into the equation of global health, to also explore complex ecological interdependencies, and the biological history of those interdependencies (Landecker 2016)—where unforeseen environmental consequences of public health and unequal material relations between microbes, humans, animals and environments are constituted as (side-)effects and future segmentations of scientific and/or medical activity.

Let me take one last COVID-19 example, which has gained some media traction recently, to exemplify what I mean: the so-called “corona litter”. In a legitimate and important attempt to stop SARS-Cov-2 infections through hygienic measures, disposable gloves and masks have become widely used in hospitals and households alike. They have also become discarded items, thrown away on streets, in parks, in forests and in rivers. Environmental activists fear that these hygienic items, and even more so single-use plastic bags, cups or plates, which have been promoted as virus safe during the pandemic, are worsening the plastic problem of our planet. 13 million tons of plastic leak into the ocean every year already, if they are not “ending up in landfills (…) or worse: in incinerators, where the neighboring communities are being inundated with dioxins and particulate emissions that harm their health”, as the environmental policy advisor Mariam Gordon has stated recently.^8^ In a similar vein, the historian Sarah Hodges has shown that hospitals, as sites of intense hygienic prescription, have long become “factories of medical garbage” (Hodges 2017), that affect populations unequally. It is unclear yet how much the massive preventive and curative use of antibiotics during the ICU care of patients with COVID-19 will impact on universal public health policies in the future, given the global rise of antimicrobial resistance (Reardon 2020).

This, too, is more-than-human public health: a public health that is always already beyond the reach of human control, entangled in a complex web of powerful scientific, economic and political more-than-human relations and “feedback loops” (Berlant 2011, p. 192). Novel infectious diseases might well amplify the environmental and medical side-effects and pollution loops of current public health measures, in the form of hygienic medical waste or antimicrobial resistance, with unequal and unknown effects on people, animals and microbes worldwide. With Landecker, I therefore see more-than-human public health as a biopolitics that is not simply sacrificial, as Keck puts, but also as a practice that is “increasingly forced to face up to the unexpected material growths of yesterday’s techniques of control, which were enacted at national or global population scale” (Landecker 2016, p. 25). Today’s material present is tomorrow’s history and biology. A more-than-human public health framework sensitizes us to the yet poorly understood, dynamic relations of long-term species interdependency. But it can also make us notice the diverse more-than-human casualties of public health interventions, and work towards alternatives.

## Footnotes

1 This topic has meant significant media coverage for Keck’s work in the COVID-19 pandemic in France, the country where he lives and works, as the government has been sharply criticized by virologists as well as infectious disease physicians for the systematic dismantling of preparedness during the last decade.

2 Keck now also directs the *Laboratoire d’anthropologie sociale* in Paris, founded by Lévi -Strauss in 1960.

3 See https://dukeupress.wordpress.com/2020/04/13/qa-with-frederic-keck-author-of-avian-reservoirs/ .

4 Sentinel chicken are unvaccinated chicken that are placed at the ends of the rows of cages in poultry farms, so that farmers and veterinarians can detect infection of livestock in farms early on. “Sentinel chickens” is the English translation for the Chinese “shaobingji” which means “whistling soldier chicken” (p. 70). The military metaphor indexes a sending of signals “from the advanced posts of the battlefield” (p. 70).

5 “Le bricoleur fait partie d’un monde entièrement signifiant, dont il combine autrement les signes pour produire de nouvelles significations“ (pp. 34-35).

6 Indeed, Cassidy herself as reflected on these parallels in a recent blogpost. See: https://www.researchprofessionalnews.com/rr-news-political-science-blog-2020-5-uk-s-coronavirus-response-repeats-the-errors-of-past-crises/

7 https://www.sciencemag.org/news/2020/06/coronavirus-rips-through-dutch-mink-farms-triggering-culls-prevent-human-infections

8 See: https://grist.org/climate/its-official-reusables-are-safe-during-covid-19/.
